# The multidisciplinary and participatory process to develop the Rubric for Learning Communities about Health Approaches

**DOI:** 10.3389/fpubh.2025.1453197

**Published:** 2025-03-05

**Authors:** Maud J. J. ter Bogt, Hilde Tobi, Christine G. J. I. van Straten, Gerard R. M. Molleman, Maria E. T. C. van den Muijsenbergh, Kirsten E. Bevelander, Gerdine A. J. Fransen

**Affiliations:** ^1^Primary and Community Care, Radboud University Medical Centre, Nijmegen, Netherlands; ^2^AMPHI Academic Collaborative Centre, Nijmegen, Netherlands; ^3^Biometris, Wageningen University & Research, Wageningen, Netherlands; ^4^Pharos, The Dutch Centre of Expertise on Health Disparities, Utrecht, Netherlands

**Keywords:** learning community, community of practice, assessment, rubric development, partnership, learn, health, health approach

## Abstract

**Introduction:**

Many Dutch municipalities implement a systems approach to promote health behavior among citizens. Learning communities (LCs) in these approaches enable stakeholders to collaborate and learn from one another. To optimize LCs, insights are needed into how LCs create knowledge and put it into action. This study aimed to describe the multidisciplinary and participatory process to develop a rubric for multidisciplinary Learning communities about health approaches.

**Methods:**

The rubric development took the form of a questionnaire, and was centred on a municipal healthy weight approach. The development consisted of three steps: (1) an iterative process involving literature and input from members and experts, (2) an expert session, and (3) qualitative and quantitative rubric reliability and usability tests.

**Results:**

Five rubric versions were developed, resulting in a final version with eight constructs to assess LC partnership experiences, learning, and action. The rubric demonstrated a relatively high reliability. The rubric’s adequate usability performance was evidenced by its high response rate, which enabled researchers to gain insights into notable findings. These findings then facilitated discussions among LC members and formulated LC adjustments.

**Discussion:**

The participative process played a crucial role in developing the rubric. LC facilitators are encouraged to apply the rubric. Future research is needed regarding the reliability and usability of the rubric in other settings.

## Introduction

Many Dutch municipalities have implemented health approaches aimed at influencing the health environment and improving health, such as healthy weight approaches to influence the obesogenic environment and reduce overweight and obesity (1). However, these approaches have had limited impact (2, 3), probably because the complexity of obesity has not been fully incorporated in them. To improve the impact of these health approaches, it is important to strengthen interprofessional collaboration, because diverse stakeholders from different domains perform different actions that ideally complement one another (4).

Five Dutch municipalities in the Gelderland-region recognized the need to strengthen health approaches by interprofessional collaboration, and therefore applied an inclusive method where multidisciplinary health approach stakeholders may learn from one another and collaborate. To enable the involvement of multidisciplinary relevant stakeholders, they started learning communities (LCs) where health approach stakeholders such as citizens, municipality policy advisors, public health service health brokers, care professionals, and practice professionals (e.g., youth worker, sports school owner, librarian) regularly get together with the aim to learn, strengthen collaboration, and align and adjust their actions (5, 6).

The LCs consisted of aspects of Professional Learning Communities, and Communities of Practice. Professional Learning Communities have been increasingly used within schools for creating a cultural educational change to continuous interactions and collaborations that increases both students’ and teachers’ learning (7–10). Both LCs and Professional Learning Communities have a common focus on learning, a shared goal, reflective dialogues, and supportive collaborative organizations (7–10). Previous studies have shown that these elements are also needed to strengthen health approaches (11, 12). Furthermore, both LCs and Communities of Practice have been increasingly used as informal networks that create opportunities for knowledge exchange, originating from the education and business sectors (12–15). Therefore, members casually share knowledge, learn and build relationships with other members with whom they do not work every day (12–15). This corroborates with the importance of cross-domain collaboration within health approaches (4) Altogether, the LCs in the current study can be defined as communities where multidisciplinary stakeholders get together during meetings to learn, collaborate, and align or adjust actions to strengthen their work.

Collaborations such as LCs are frequently used and assessed in monodisciplinary collaborations (5, 7, 13, 16), but there is limited literature about public health LCs that require multidisciplinary collaborations (4), while LCs are becoming more popular in multidisciplinary collaborations in health approaches (17). LCs may lead to output regarding individual and group learning (5). Group learning is implicit or explicit knowledge gained from the interaction of LC members’ health approach experiences (18, 19). Ideally, this knowledge creation is applied, meaning that tasks are added, erased, or performed differently. Yet, many studies report that translating knowledge into action is difficult and depends, for example, on stakeholder involvement and adequate tailoring to the local context (20).

Repeatedly assessing LCs may gain insights into whether and how LC members create knowledge and apply it in practice. These insights assist LC members to make necessary adjustments in LC meetings, such as regarding the LC meeting agenda and involved LC members. This may enhance LC effectiveness regarding both knowledge creation and its practical application (21). This importance is emphasized by the existence of multiple Professional Learning Community rubrics, such as The Professional Learning Community Assessment-Revised, which is a recognized instrument for assessing Professional Learning Communities in schools (22–24). However, these rubrics do not apply to the public health LC context as they are not matched to multidisciplinary stakeholders, health approaches, or the Dutch context (25). Still, within our context, some relevant non-validated rubrics are available (26–31), but these focus on either collaboration, learning, or action rather than its combination. Therefore, it is needed to develop a “Rubric for multidisciplinary learning communities about health approaches.” To do this, a better understanding of the usefulness of the currently available non-validated rubrics within our contexts is needed (26–31), additional rubric aspects need to be developed, and it needs to be examined how these can be operationalized to create an effective rubric.

A multidisciplinary approach is required to develop a rubric, because LCs are complex, dynamic, and consist of multiple key characteristics originating from different disciplines (5, 21). Therefore, the expertise of LC experts is required. Furthermore, LC members’ experiences are essential, as the rubric aims to assess their experiences in terms of learning and action, and to assist them in adjusting LCs when required. Participatory methods in which LC members and experts support rubric development are thus crucial. Participatory methods were not commonly used in previous rubric development studies, but studies that included participatory approaches showed promising results (32). Furthermore, participatory methods for health intervention development contributed to shared responsibility, relevant developed health interventions, and sustained intervention effects (33, 34). Research is thus needed to gain insights into the multidisciplinary and participative process to develop a valid rubric with adequate usability performance. Therefore, this study aims to describe a multidisciplinary and participatory process to develop a rubric to repeatedly assess LC partnership experiences, learning, and action among multidisciplinary LCs about health approaches. Sub-aims include understanding what needs to be assessed, the process of transforming this into a rubric, how rubric data can provide feedback to LC members, and how this may help LC members in formulating adjustments to further facilitate learning and aligned actions.

## Materials and methods

### Study setup

Across five municipalities in the Netherlands (Gelderland region), one LC of two adjacent municipalities and one LC of three adjacent municipalities were implemented in 2021 with the aim to create more effective health approaches. More specifically, these LCs focused on a healthy weight approach. A healthy weight approach refers to all elements (e.g., activities, facilities, and policies) that (in)directly promote physical activity and a healthy dietary intake, and reduce citizens’ sedentary behavior (11). LC members comprised a multidisciplinary and relatively stable group of 12 to 20 healthy weight approach stakeholders, including municipal policy advisors (one per municipality), municipal health service health brokers (one per municipality), healthcare professionals such as general practitioners and dietitians, and individuals engaged in practical roles such as welfare workers, sports school owners, and citizens. As citizens are the target group of the healthy weight approach, citizens who wanted to contribute to more effective healthy weight approaches were recruited via advertisement posters displayed in public spaces, as described elsewhere (35, 36). Members were invited for every LC meeting. The number of present members per LC meeting varied among both LCs between 7 and 17, as further explained elsewhere (35). At least one LC member from each municipality was present at every meeting.

LCs meet in an environment where the exchange of knowledge, inspiration, reflection, and plan creation is put into practice by applying the observe–reflect–plan–act cycle (37). This meant that, between LC meetings, LC members observed health approaches from their own perspective and put their plans into action, while researchers conducted studies on health approaches (11, 36). During LC meetings, the researchers pitched the research results, and the LC members pitched action updates and/or health approach observations based on personal experiences. Next, the LC members reflected upon these observations and created plans by applying creative and participatory methods (38). Each LC meeting ended by bundling new plans on a dynamic learning agenda (28). The first three LC meetings each took four hours every six months. Subsequent meetings took 2.5 hours every three months. More information about the LCs is elaborated on elsewhere (35).

### Procedure and data analysis

**Figure 1:**
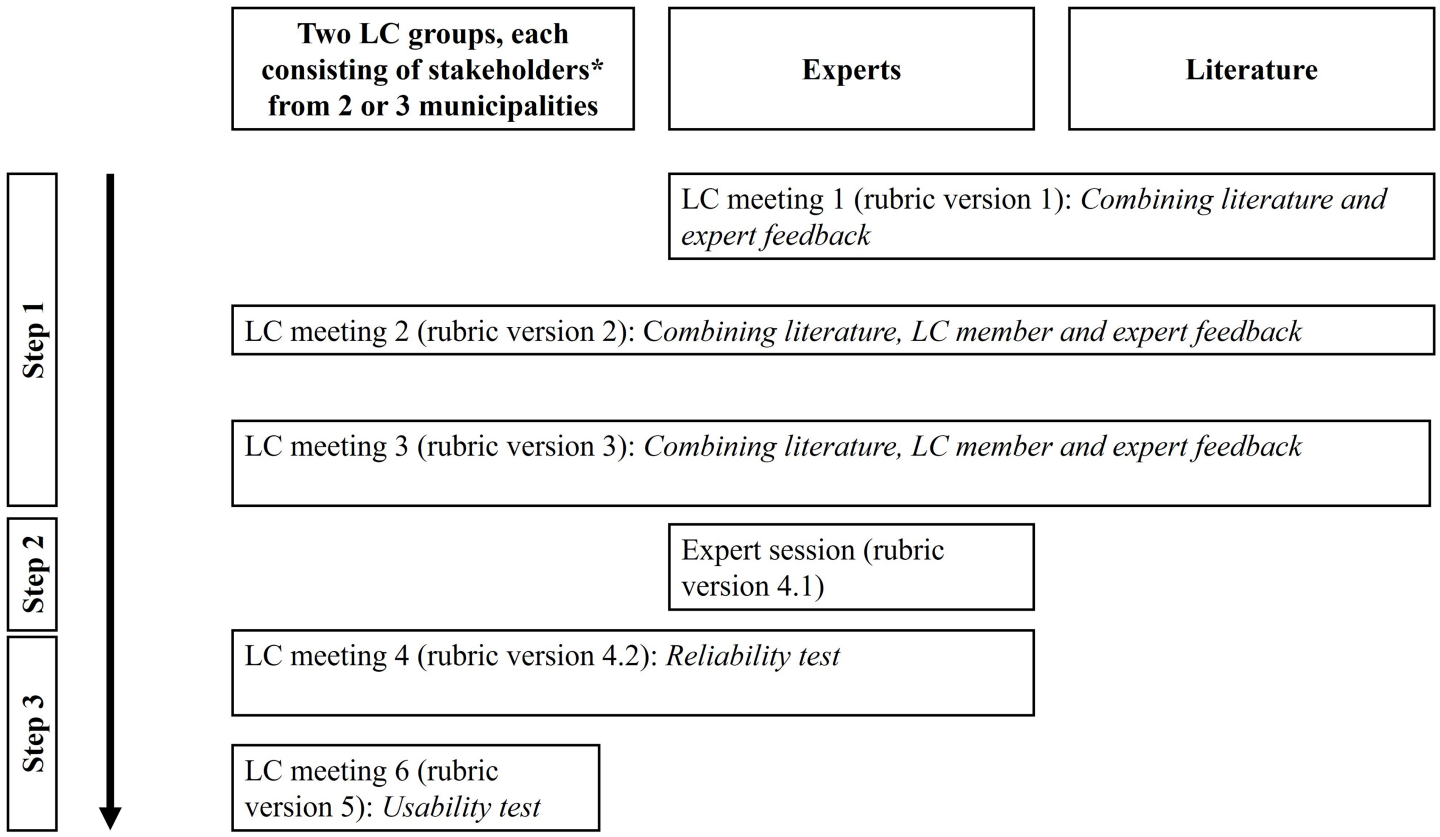
Schematic timeline of rubric development, including the involvement of LCs, experts, and literature. *Stakeholders include municipal policy advisors, municipal health service health brokers, healthcare professionals, and professionals engaged in practical roles, such as welfare workers, and citizens.

The rubric development was a participatory, iterative three-step process that took place across two LCs during the same period, combining practical experience-based (multidisciplinary LC members) and knowledge-based feedback (multidisciplinary experts, and literature) (39) (Figure 1). The different rubric versions led to a rubric questionnaire that aimed to enable repeated assessment of each specific LC meeting’s (1) LC partnership experiences, (2) learning, and (3) action. The rubric was applied among LC members every six months after an LC meeting.

#### Step 1: iterative process combining literature and LC member and expert feedback

Step 1 included an iterative process in which the main researcher (MB) combined feedback from literature, LC members, and experts throughout LC meetings 1 to 3 (October 2021 to September 2022). More specific, desk research was performed to identify literature about existing rubrics or rating matrices. Next, literature relevance regarding LC partnership experiences, learning and action, and required contextual adaptations to Dutch LCs about health approaches were discussed during expert consultations. Next to the research team (KB, GF, GM, and MM), the main author (MB) invited additional Dutch experts with relevant experiences and expertise (AW, HT, LF, and WK). In total, eight scientific experts participated, with expertise in LC partnership experiences (*n* = 2), learning (*n* = 2), collective action (*n* = 3), or questionnaire construction (*n* = 1). Furthermore, LC members present at LC meetings 1, 2, or 3 were asked to fill in the rubric after the LC meeting. If necessary, a reminder to fill in the rubric was sent at the end of the day, and a second reminder the day after. These rubric responses were used as LC member rubric feedback.

#### Step 2: expert session

Step 2 comprised an expert session, organized apart from the LCs (January 2023). Prior to the expert session, experts were asked to sort the closed-ended items of the last rubric version of Step 1. These individual assignments were bundled as the starting point, as they indicated current consensus and inconsistencies among experts. The expert session aimed to classify closed-ended items in pilot constructs to assess LCs, define the pilot constructs, and discuss the face validity of items until consensus was reached, resulting in rubric version 4.1. The eight LC experts involved in Step 1 participated, supplemented with one non-scientific facilitator of other comparable LCs in other municipalities (HB). Furthermore, one developer of a relevant tool found in the literature (28) was individually consulted afterward to check construct completeness, as the expert (BM) was not available during the expert session.

#### Step 3: rubric reliability and usability test

Step 3 covered rubric reliability and usability tests based on quantitative and qualitative methods. LC members present at LC meeting 4 (January 2023, *n* = 24) or 6 (June 2023, *n* = 13) were asked to fill in the rubric after the LC meeting. If necessary, a reminder to fill in the rubric was sent at the end of the day, and a second reminder the day after. The consistency between items within a construct was determined, which is further referred to as rubric reliability. For all items belonging to the same pilot construct, a split half reliability coefficient was calculated in R (version 2022.02.1) using data from LC meeting 4. A split half reliability coefficient above 0.7 was considered as good reliability (40), and these sets of items were considered final for the respective constructs. All pilot constructs for which the items had a split half reliability coefficient below 0.7 were individually discussed with experts in the corresponding field, until the experts and the first author decided that the set of items for that construct was final. Finally, definite split-half reliability coefficients were calculated again for the final constructs, based on the data from LC meeting 6. The smallness of the participant sample precluded other analyses, such as Confirmatory Factor Analysis (41).

Applying the concept of usability to our rubric suggested adequate usability performance when (1) the instrument had an adequate response rate, (2) researchers were able to formulate notable rubric findings accepted by LC members and the LC facilitator, and (3) these rubric findings helped LC members formulate how they could adapt the LC accordingly (42–45). To gain insights into rubric usability, the rubric results of LC meetings 2, 3, and 4 were discussed with the LC members during LC meeting 6 (June 2023), resulting in rubric version 5. To do this, the data from LC meetings 2, 3, and 4 were analyzed in R (version 2022.02.1). LC meeting 1 was excluded as it entailed a focus group rather than an actual LC meeting, and the constructs were largely incomplete because of the stage of the rubric development process. For all three LC meetings, the median and the interquartile range (minimum, maximum) were calculated per final construct for both LC groups, which were visualized using a boxplot. Supplementary materials 1, 2 describe, respectively, how different rubric versions and different answer scales were processed to facilitate comparison over time (46, 47).

## Results

### Step 1: iterative process combining literature and LC member and expert feedback

Desk research identified relevant literature about LCs applicable to our context. These included for partnership experiences the Coordinated Action Checklist (26), for learning the Boundary Crossing Theory (27, 48) and Value Creation (49), and for action the Collective Impact Principles (12, 29–31) and Reflexive Monitoring (28); which was used in rubric development versions 1 to 3 (Supplementary material 3).

For the first LC meeting, rubric version 1 was based on items from the Coordinated Action Checklist, because it adequately assesses partnership experiences (26). Furthermore, five items about health approach adaptations based on learning were self-formulated (MB, GF, KB, GM, and MM), as this was the overall project goal, but no items were found in the literature. These items were measured on a 6-point scale (no, definitely not; no, I do not think so; maybe; yes, I think so; yes, definitely; I do not know/not applicable) (26).

For the second LC meeting, rubric version 2 was complemented with items on action originating from Reflexive Monitoring (28) as, according to experts, it was essential to assess the action concept (GM, GF, and KB). Furthermore, items regarding Boundary Crossing Theory were included (27, 48), because experts on learning (LF and WK) indicated that these items would further substantiate the assessment of learning. These items were measured on a 1 (completely disagree) to 10 (completely agree) answer scale. Finally, two open-ended questions about learning in terms of value creation were included (49) on the advice of learning experts (LF and WK), as this information was needed to assess LCs adequately, and LC members desired open-ended questions.

For the third LC meeting, rubric version 3, which was the last version prior to the expert meeting, was complemented with items based on Collective Impact Principles (12, 29–31), because experts (GM, GF, and KB) believed that this would further substantiate the action concept. These items were measured on a 1 (completely disagree) to 10 (completely agree) answer scale. Furthermore, the Coordinated Action Checklist item regarding equivalence (26) was excluded as LC members and experts (AW, HT, KB, and GF) interpreted it differently and agreed that it did not accurately reflect the partnership. Moreover, open-ended questions were included as LC members wanted to be able to explain experiences. Among these, one question was added to complete the aspect of learning in terms of Value Creation (49) (Supplementary material 4).

### Step 2: expert session

Experts excluded overlapping or irrelevant items, reformulated or split items to increase understandability, and added items to complete constructs (Supplementary material 3). The expert session resulted in 50 items grouped into seven pilot constructs: perceived cooperation LC, involvement, learning from one another, keep learning, LC output, intentions, and network composition (Supplementary material 3). Furthermore, all answer scales were changed to an 11-point answer scale with 1 (completely disagree) to 10 (completely agree), and the additional “I do not know/not applicable” option, because LC members and experts desired identical answer scales for item comparison, and perceived that the previously used 5-point Likert scale was more difficult to use. This resulting rubric was version 4.1 (pilot constructs).

### Step 3

#### Rubric reliability test

Most rubric version 4.1 constructs showed adequate reliability. The pilot constructs learning from one another, keep learning, LC output, intentions, and network composition had a split half reliability coefficient above 0.7. The involvement and perceived cooperation LC pilot constructs had split-half reliability coefficients of 0.54 and 0.63, respectively, and were therefore discussed during individual consultations with LC partnership experience experts (AW, HB, and GF). Subsequently, the involvement construct was split into involvement LC and involvement approach, and one item was reincluded from rubric version 3 (Supplementary material 3). Two items were excluded from the perceived cooperation LC construct (Supplementary material 3). Split-half reliabilities of these three adapted constructs increased slightly, but two constructs stayed below 0.7 (Supplementary material 5). All individually consulted experts and the main author were confident about these three constructs and their definitions, resulting in rubric version 4.2 (final constructs; Table 1), which consisted of 49 closed-ended items divided over the final eight constructs (Supplementary material 3).

**Table 1:** Final constructs and two example items (full overview in Supplementary material 5).

Final construct	Two example items
Perceived cooperation LC^1^	The relationships among the LC members are strong. The LC members are open in their communication.
Involvement approach^1^	I feel that strengthening the healthy weight approach is urgent. I want to contribute to a change within the healthy weight approach, even if it requires a personal change and a change in my organization.
Involvement LC^1^	I feel involved in the LC. I create goodwill and involvement for the LC within my organization/department.
Learning from one another^2^	The LC made me realize my knowledge gaps regarding strengthening the healthy weight approach. The LC uses input from various LC members to gather new solutions.
Keep learning^2^	I want to continue using the LC method after the project has ended. I feel responsible for reflecting on information about the healthy weight approach.
LC output^2^^,^^3^	In the LC, an increasingly concrete shared goal and vision is developed. The LC meeting helps me make the necessary adjustments in the current healthy weight approach.
Intentions^3^	I will apply what I have learned in daily practice. The LC meeting helped me generate new ideas about the healthy weight approach.
Network composition^1^^,^^2^^,^^3^	The LC involves the correct partners to achieve its purpose. The LC members have good contact with collaboration partners outside of the LC.

1 LC partnership experiences.

2 Learning.

3 Action.

#### Rubric usability test

Three aspects stood out regarding the final construct usability. First, the rubrics had an adequate response, as they were completely filled in by 91.7 to 100% of the present LC members in LC meetings 2, 3, and 4, representing 70.0 to 94.4% of the LC members.

Second, to gain insights into notable rubric findings from LC meetings 2, 3, and 4, researchers (MB and CS) discussed per LC group the predominant results regarding (1) high or low construct scores compared with other construct scores obtained in the same meeting, (2) increasing or decreasing construct scores over time, and (3) differences in construct scores between LC groups. This resulted in the formulation of three to five rubric findings per LC (Table 2). These findings were accepted by the LC facilitator and researchers present at the LC meetings. Furthermore, the LC facilitator and researchers indicated that the rubric findings enabled them to formulate more specific positive and improvement points for LCs than LC observations alone. A video was created to demonstrate the notable rubric findings to the LC members during LC meeting 6. These rubric findings were accepted by the LC members.

**Table 2:** Notable rubric findings per LC as provided during LC meeting 6.

Notable rubric findings LC group A^1^	Notable rubric findings LC group B^1^
Involvement LC scored relatively highly compared with other constructs scores, but declined considerably from October 2022 to January 2023.	Involvement LC scored relatively highly during January 2022 compared with other construct scores. However, it decreased considerably to October 2022 and increased slightly again to January 2023.
Learning from one another declined slightly from October 2022 to January 2023, resulting in a medium score compared with other construct scores.	Keep learning scored highly during January 2022 but decreased considerably afterwards becoming a medium construct score.
Intentions decreased slightly from October 2022 to January 2023, becoming the lowest construct score.	Intentions increased slightly from January 2022 to October 2022, yet decreased considerably to January 2023, becoming a medium construct score compared to other construct scores.
LC output increased slightly from January 2022 to October 2022 and stayed stable until January 2023.	
Perceived cooperation LC recorded a medium score during October 2022 compared with other construct scores yet declined considerably during January 2023.	

1 High, medium, or low construct scores refer to a comparison of the construct of interest with other construct scores.

Third, the notable rubric findings (Table 2) enabled discussion among LC members during LC meeting 6, resulting in the formulation of LC meeting adjustment wishes. In both LCs, all LC members present participated in this discussion. LC members initiated further explanations and interpretations regarding the notable rubric findings. For example, LC members of LC group A indicated that formulating action plans and providing action plan updates during LC meetings limited LC participation by some health approach partners, which may explain the decline in LC involvement and intentions. These notable rubric findings enabled LC members to formulate both the LC elements that they wanted to retain and those that they wanted to adjust in future LC meetings. For example, to stimulate involvement in LC, learning from one another, and network composition, LC members of LC group A agreed to focus on current health approach blind spots at the next LC meeting and create a second LC shell to involve more health approach stakeholders.

#### Final rubric

After LC meeting 6, one rubric iteration was made, resulting in rubric version 5, which was the final rubric (Table 1; Supplementary materials 4, 5). As LC members perceived several items as hard to answer during the first LC meetings as the answer would become clear only in the future, these items were scored in the middle of the answer scale (5 or 6) by some LC members, whereas others scored them “I do not know/not applicable.” Therefore, on the 11-point answer scale, the “I do not know/not applicable” answer category was reformulated to 0 (absent/not applicable) (Supplementary material 3) to facilitate similar usage by LC members. Data obtained from LC meeting 6 suggested a near-excellent reliability, with all split-half coefficients larger than 0.90 (Supplementary material 5), suggesting that the answer scale iteration was easy to use.

## Discussion

To the best of our knowledge, this is the first study to use a participatory process to develop a rubric for multidisciplinary LCs about health approaches. Our study showed that LC partnership experiences, learning, and action are important topics that can be assessed by eight constructs. Practical experiences and knowledge-based approaches were combined through an iterative process by incorporating input from the literature, LC members, and LC experts. Both multidisciplinary members and experts continuously made contributions to rubric iterations, demonstrating their willingness and capability to participate actively in the rubric development. Furthermore, the input from both LC members and experts complemented each other, highlighting that the inclusion of both groups, rather than just one, enhances rubric development. This participatory process proved to be feasible and effective in facilitating rubric development.

Our findings from Step 3 suggested that the rubric had adequate usability, as (1) the instrument achieved a sufficient response rate, (2) researchers were able to formulate noteworthy rubric findings that were accepted by LC members and the LC facilitator, and (3) these rubric findings helped LC members to formulate adaptations for the LC as needed. The instrument can help LC members to individually reflect on specific constructs and subsequently reflect together in plenaries on outstanding construct scores, as reported as useful in previous studies (50, 51). Moreover, other studies about the two LCs involved in this study suggested that the LCs gained learnings and took actions in relation to the healthy weight approach, suggesting that the LCs were functional (35, 52). For example, in the closed-ended rubric questions members indicated how much LC members learned from one another, and in the open-ended rubric questions members indicated several examples about what they have learned. More specific, during LC meeting 6 and the corresponding rubric results, the members indicated that they learned, among others, about the existence of a movement toolbox that can be used during sports lessons. Subsequently, some members formulated follow-up actions to explore the whether they can apply the toolbox in their current practices. Therefore, all multidisciplinary LCs about health approaches are recommended to use the rubric repeatedly. LC facilitators who want to apply the rubric are advised to determine their assessment aim together with LC members and subsequently determine together the constructs that they want to evaluate, and how frequently. Future research could gain insights into how the rubric can be used among LCs that focus on (public health) contexts other than health approaches by contextualizing the rubric. For example, slight reformulations such as replacing the words, healthy weight approach, by words that describe the public health context in which the LC of interest operates. Furthermore, future research could gain insights into the applicability of this rubric development process to different cultural contexts. The rubric seemed to have sufficient reliability, as evidenced by split-half reliability coefficients (40) and expert consensus (53). In addition, future research involving multiple LCs that utilize the rubric is recommended to assess its sensitivity to change (39). For instance, longitudinal mixed models could be used to study the differences in construct scores that can be considered small, medium, and large changes over time.

The rubric can be used for both research and practice purposes that aim to optimize LC meetings, where LC members fill in the rubric and its results are plenarily discussed to make necessary adjustments, provided that users adhere to four boundary conditions. First, the rubric should be used along with regular LC evaluation signals; this is in line with an instrument used in step 1 of the rubric development process (26). To prevent missing important LC experiences that are likely to be unsaid or unnoticed, the LC facilitator and LC members ideally also observe LC signals, such as (non)verbal communication during LC meetings, and LC members ideally also reflect on these signals in addition to the rubric results. Second, the rubric results should be interpreted with LC members, as also indicated in comparable contexts (26). Therefore, a plenary discussion to reflect on notable rubric findings from an LC meeting is required to formulate adjustments. Third, when the rubric is used for LC optimalization, not all factors that underly the constructs are covered, such as LC members’ individual time investment (54). Therefore, the LC facilitator should facilitate the plenary discussion of rubric findings so that these specific factors may be addressed by LC members when needed. Fourth, the LC facilitator should enable LC adjustments desired by LC members, as also reported previously (38). Altogether, the described process may help LC members in formulating LC (meeting) adjustments that may further facilitate learning and aligned action in LCs.

### Strengths and limitations

A significant strength of the present study is that the rubric versions were developed and investigated in real-life LCs as an integral part of ongoing health approach programs (32). This real-life setting increased the ecological validity of our findings with respect to the multidisciplinary participatory procedure of instrument development and testing. Furthermore, the main author played dual roles by being responsible for both rubric development and facilitating the LCs, as is common in action research. The dual roles can be considered both a strength and a limitation of the study. On the one hand, this facilitated the practical application of the rubric during LC meetings and co-development with LC members. However, LC members may have been more likely to complete the rubric or provide socially desirable responses. As the rubric was developed with two LCs, it was only conducted among a limited number of participants. Furthermore, as both LCs were about health approaches in the same region of the Netherlands, limited insights were gained regarding the applicability of the rubric to other LCs and the rubric development process to other cultural contexts.

## Conclusion

The multidisciplinary, participatory, and iterative process in which literature was combined with input from LC members and experts yielded a usable and reliable rubric. Using eight constructs, the rubric for health approaches assesses LC partnership experiences, learning, and action. More research is needed on rubric performance and the rubric development process in other contexts.

## Data Availability

The original contributions presented in the study are included in the article/Supplementary material, further inquiries can be directed to the quality team of our department of primary care (kwaliteitsteam.elg@radboudumc.nl).
