# Supplementary material for: The multidisciplinary and participatory process to develop the Rubric for Learning Communities about Health Approaches
Source: Front Public Health. 2025 Mar 5;13:1453197. doi: 10.3389/fpubh.2025.1453197 (PMC11919888; doi:10.3389/fpubh.2025.1453197)
Supplement: Supplementary file 1 [file Table_1.docx]

## **Supplemental material 1: Processing of different construct compositions (missing or reformulated items) to facilitate comparison over time**

| **LC meeting 6**  **Final construct** | **Processing to facilitate comparison over time^1^** | **LC  meeting 4** | **LC meeting 3** | **LC meeting 2** |
| --- | --- | --- | --- | --- |
| **Perceived cooperation LC** (10 items) | Mean score normalized for number of items (8–10) per participant  Median score per LC group | 1 item reformulated |  | 2 items missing |
| **Involvement LC**  (4 items) | Mean score normalized for number of items (3–4) per participant  Median score per LC group |  | 1 item missing | 1 item missing |
| **Involvement approach** (3 items) | Median score per LC group | 1 item reformulated |  |  |
| **Learning from one another** (8 items) | Mean score normalized for number of items (7–8) per participant  Median score per LC group | 7 items reformulated | 1 item missing | 1 item missing |
| **Keep learning** (5 items) | Mean score normalized for number of items (2–5) per participant  Median score per LC group | 1 item split into 3 items | 3 items missing | 3 items missing |
| **LC outputs** (9 items) | Mean score normalized for number of items (7–9) per participant  Median score per LC group | 1 item reformulated |  | 2 items missing |
| **Intentions** (3 items) | Median score per LC group | 3 items reformulated |  |  |
| **Network composition** (7 items) | Mean score normalized for number of items (6–7) per participant  Median score per LC group | 3 items reformulated |  | 1 item missing |
| Presented at LC meeting 6: 3–5 rubric findings per LC group | | | | |

^1^ As rubric versions 1 to 3 had missing or differently formulated items compared with MI version 4.2, construct scores were corrected for previous rubric versions. These corrections were determined by normalizing the mean score for number of items per participant. Further, as the rubric of LC meeting 4 consisted of rubric version 4.1 followed by rubric version 3, effects of reformulations were estimated by comparing median scores of originally formulated and reformulated items. This was taken into account when formulating rubric findings.
