# Supplementary material for: The multidisciplinary and participatory process to develop the Rubric for Learning Communities about Health Approaches
Source: Front Public Health. 2025 Mar 5;13:1453197. doi: 10.3389/fpubh.2025.1453197 (PMC11919888; doi:10.3389/fpubh.2025.1453197)
Supplement: Supplementary file 2 [file Table_2.docx]

## **Supplemental material 2: Processing different answer scales to facilitate comparison over time**

The I don’t know/not applicable answer option was replaced by the value 0. As some items of rubric versions 1 to 3 were measured on a 6-point answer scale, these were converted to an 11-point answer scale based on X_11-point score_ = (X_6-point score_ – 1) * (10 – 1) / (5 – 1) + 1 (46, 47).

| **Original 6-point score** | **Converted 11-point score** |
| --- | --- |
| 0 | 0 |
| 1 | 1 |
| 2 | 3.25 |
| 3 | 5.5 |
| 4 | 7.75 |
| 5 | 10 |
