# Supplementary material for: The multidisciplinary and participatory process to develop the Rubric for Learning Communities about Health Approaches
Source: Front Public Health. 2025 Mar 5;13:1453197. doi: 10.3389/fpubh.2025.1453197 (PMC11919888; doi:10.3389/fpubh.2025.1453197)
Supplement: Supplementary file 3 [file Table_3.docx]

**Supplemental material 3: Overview of the rubric development in terms of item iterations, construct development, and professional Learning Community (LC) meeting at which it was used**

| **Rubric** **versions** | **LC meeting** | **Included items** | **Excluded items** | **Other rubric item iterations** | **Answer scale iterations** | **Constructs** |
| --- | --- | --- | --- | --- | --- | --- |
| **Step 1: Iterative process combining literature, LC member and expert feedback** | | | | | | |
| Rubric version 1   - 37 closed-ended items | 1 (n = 25) | - 32 items (Coordinated Action Checklist) - 5 items (formulated regarding learning monitoring and learning together) | X | X | - [All items] 6-point: no, definitely not; no, I don’t think so; maybe; yes, I think so; yes, definitely; I don’t know/not applicable | No constructs determined |
| Rubric version 2   - 61 closed-ended items - 2 open-ended items | 2 (n = 30) | - 12 items (Reflexive Monitoring) - 12 items (Boundary Crossing Theory) - 2 open-ended items (Value Creation) | X | X | - [37 items] 6-point: no, definitely not; no, I don’t think so; maybe; yes, I think so; yes, definitely; I don’t know/not applicable. - [24 items] 10-point: 1 completely disagree to 10 completely agree | No constructs determined |
| Rubric version 3   - 70 closed-ended items - 8 open-ended items | 3 (n = 22) | - 10 items (Collective Impact Principles) - 5 open-ended items to enable explanations (2 about tips and tops for next LC meetings, 3 general remark fields) - 1 open-ended item (Value Creation) | - 1 item about equivalence (Coordinated Action Checklist), because of different interpretations not accurately reflecting partnership experiences and being excluded from the original questionnaire | X | - [36 items] 6-point: no, definitely not; no, I don’t think so; maybe; yes, I think so; yes, definitely; I don’t know/not applicable. - [34 items] 10-point: 1 completely disagree to 10 completely agree | No constructs determined |
| **Step 2: Expert session** | | | | | | |
| Rubric version 4.1 (pilot constructs)   - 50 closed-ended items - 8 open-ended items | 4 (n = 24) | - 1 item about engagement in the LC for the involvement LC construct - 1 item about understanding the steps needed to strengthen the HA for the learning from one another construct | - 24 items, mostly because of overlapping items or insufficiently corresponding to a construct | - 16 items reformulated to increase understandability - 1 item split into 3 items, as the original item covered three different aspects | - [All items] 11-point: 1 completely disagree to 10 completely agree, and I don’t know/not applicable | 7 constructs: perceived cooperation LC (12 items), involvement (6 items), learning from one another (8 items), keep learning (5 items), LC output (9 items), intentions (3 items), and network composition (7 items) |
| **Step 3: rubric reliability and usability test** | | | | | | |
| Rubric version 4.2 (final constructs)   - 49 closed-ended items - 8 open-ended items | 4 (n = 24) | - To complete the involvement approach construct, one item that had been excluded to achieve rubric version 4.1 was reincluded | - Perceived cooperation LC construct: two items regarding trust and critically discussing own ideas were excluded, as these transcended the construct | X | X | The involvement construct was split into involvement LC (4 items) and involvement approach (3 items), and perceived cooperation (10 items) was finalized, resulting in 8 constructs (Supplemental material 5) |
| Rubric version 5   - 49 closed-ended items - 8 open-ended items | 6 (n = 13) | X | X | X | - [All items] 11-point: 0 (absent/not applicable) and 1 completely disagree to 10 completely agree | X |
