# Supplementary material for: The multidisciplinary and participatory process to develop the Rubric for Learning Communities about Health Approaches
Source: Front Public Health. 2025 Mar 5;13:1453197. doi: 10.3389/fpubh.2025.1453197 (PMC11919888; doi:10.3389/fpubh.2025.1453197)
Supplement: Supplementary file 4 [file Table_4.docx]

**Supplemental material 4: Rubric version 5**

**Evaluation learning community [municipality names]**

You are a member of the learning community [municipality names]. This learning community is a collaboration between stakeholders involved in the healthy weight approach in these municipalities. The learning community is the group with which you attended the meeting today as well as the appointed members who could not attend. This questionnaire is about your personal experiences and impressions of the whole learning community as you perceive it now. This questionnaire is about your opinions, not about facts.

What is your (work) e-mail address where I may reach you?…………………………………………………………………………....…….

What did you like about the learning community? Consider, for example, the format, location, interactions, and times.

(optional*)*
…………………………………………………………………………………………………………………………………………………………….…………………………………………………………………………………………………………………………………………………………….…………………………………………………………………………………………………………………………………………………………….…………………………………………………………………………………………………………………………………………………

What could be improved about the learning community in the next session? Consider, for example, the format, location, interactions, and times.

(optional*)*
…………………………………………………………………………………………………………………………………………………………….…………………………………………………………………………………………………………………………………………………………….…………………………………………………………………………………………………………………………………………………………….…………………………………………………………………………………………………………………………………………………

The evaluation consists of five parts: engagement, collaboration within the learning community, learning from the learning community, learning community yields, intentions and actions after the learning community. Fifty-three statements follow. Please indicate the extent to which you disagree or agree with the statements below. Do so by giving a rating between 1 (completely disagree) and 10 (completely agree).

|  | |  | | **0 (absent / not applicable)** | | **1 (strongly disagree)** | **2** | **3** | **4** | **5** | **6** | | **7** | | **8** | | **9** | **10 (strongly agree)** | | |
| --- | --- | --- | --- | --- | --- | --- | --- | --- | --- | --- | --- | --- | --- | --- | --- | --- | --- | --- | --- | --- |
|  |  | | **Involvement** | | | | | | | | |  |  |  |  |  |  | |  |  |
|  | | I benefit my position/organization directly by participating in the learning community. ^1^ | |  |  |  |  |  | | | |  |  |  |  |  |  | |  |  |
|  | | I feel that strengthening the healthy weight approach is urgent. ^2, a^ | |  |  |  |  |  | | | |  |  |  |  |  |  | |  |  |
|  | | I feel involved in the learning community. ^1, a^ | |  |  |  |  |  | | | |  |  |  |  |  |  | |  |  |
|  | | I create goodwill and involvement for the learning community within my organization/department. ^1^ | |  |  |  |  |  | | | |  |  |  |  |  |  | |  |  |
|  | | I want to contribute to a change within the healthy weight approach, even if it requires a personal change and a change in my organization. ^2^ | |  |  |  |  |  | | | |  |  |  |  |  |  | |  |  |
|  | | I believe that most learning community members feel engaged in the learning community. ^b^ | |  |  |  |  |  | | | |  |  |  |  |  |  | |  |  |
|  | | I feel very involved into searching for solution to strengthen the healthy weight approach.^2, b^ | |  |  |  |  |  | | | |  |  |  |  |  |  | |  |  |
|  |  | | **Collaboration within the learning community** | | | | |  | | | |  |  |  |  |  |  | |  |  |
|  | | I am satisfied with the input of all learning community members. ^1^ | |  |  |  |  |  | | | |  |  |  |  |  |  | |  |  |
|  | | I involve healthy weight approach partners that are not learning community members in what I learn. ^2, b^ | |  |  |  |  |  | | | |  |  |  |  |  |  | |  |  |
|  | | The learning community meeting encourages me to reach out to relevant healthy weight approach partners. ^2^ | |  |  |  |  |  | | | |  |  |  |  |  |  | |  |  |
|  | | The learning community meeting stimulates me to purposely collaborate with other stakeholders in healthy weight approach.^2^ | |  |  |  |  |  | | | |  |  |  |  |  |  | |  |  |
|  | | The learning community members cooperate well with one another. ^1^ | |  |  |  |  |  | | | |  |  |  |  |  |  | |  |  |
|  | | The relationships among the learning community members are strong.^2, a^ | |  |  |  |  |  | | | |  |  |  |  |  |  | |  |  |
|  | | The learning community members deal with conflicts constructively.^1^ | |  |  |  |  |  | | | |  |  |  |  |  |  | |  |  |
|  | | The learning community members have regular contact to keep one another informed and inspired. ^2,a^ | |  |  |  |  |  | | | |  |  |  |  |  |  | |  |  |
|  | | The learning community members are willing to compromise.^1^ | |  |  |  |  |  | | | |  |  |  |  |  |  | |  |  |
|  | | The learning community members are open in their communication.^1^ | |  |  |  |  |  | | | |  |  |  |  |  |  | |  |  |
|  | | The learning community members know how to find one another when something needs to be done.^1^ | |  |  |  |  |  | | | |  |  |  |  |  |  | |  |  |
|  | | Members of the learning community actively listen to one another.^1^ | |  |  |  |  |  | | | |  |  |  |  |  |  | |  |  |
|  | | The learning community functions well (structure, methods).^2^ | |  |  |  |  |  | | | |  |  |  |  |  |  | |  |  |
|  | | The learning community involves the correct partners to achieve its purpose.^1^ | |  |  |  |  |  | | | |  |  |  |  |  |  | |  |  |
|  | | The learning community members are willing to include other healthy weight approach stakeholders in the learning community meeting over time.^1^ | |  |  |  |  |  | | | |  |  |  |  |  |  | |  |  |
|  | | The learning community meeting helps to maintain the involvement of various healthy weight approach stakeholders.^1^ | |  |  |  |  |  | | | |  |  |  |  |  |  | |  |  |
|  | | The learning community members have good contact with collaboration partners outside of the learning community.^1^ | |  |  |  |  |  | | | |  |  |  |  |  |  | |  |  |

Space for explanation

…………………………………………………………………………………………………………………………………………………………….…………………………………………………………………………………………………………………………………………………………….…………………………………………………………………………………………………………………………………………………………….…………………………………………………………………………………………………………………………………………………

| **Learning from the learning community** | |  | **0 (absent / not applicable)** | | **1 (strongly disagree)** | | | **2** | **3** | **4** | **5** | **6** | **7** | **8** | **9** | **10 (strongly agree)** | |
| --- | --- | --- | --- | --- | --- | --- | --- | --- | --- | --- | --- | --- | --- | --- | --- | --- | --- |
|  | The learning community made me realize my knowledge regarding strengthening the healthy weight approach.^2^ |  |  |  | |  |  | | |  |  |  |  |  |  | |  |
|  | The learning community made me realize my knowledge gaps regarding strengthening the healthy weight approach^2^ |  |  |  | |  |  | | |  |  |  |  |  |  | |  |
|  | The learning community encourages me to share my opinion with others.^2^ |  |  |  | |  |  | | |  |  |  |  |  |  | |  |
|  | The learning community encourages me to learn from others.^2^ |  |  |  | |  |  | | |  |  |  |  |  |  | |  |
|  | I now have a better understanding of the next steps that I can take to strengthen the healthy weight approach than I did before the learning community meeting.^b^ |  |  |  | |  |  | | |  |  |  |  |  |  | |  |
|  | The people that I work with also learn from my involvement in the learning community.^2^ |  |  |  | |  |  | | |  |  |  |  |  |  | |  |
|  | I want to continue using the learning community method after the project has ended.^1^ |  |  |  | |  |  | | |  |  |  |  |  |  | |  |
|  | I feel responsible for gathering information about the healthy weight approach.^c^ |  |  |  | |  |  | | |  |  |  |  |  |  | |  |
|  | I feel responsible for reflecting on information about the healthy weight approach.^c^ |  |  |  | |  |  | | |  |  |  |  |  |  | |  |
|  | I feel the need to do something with the newly acquired information about the healthy weight approach.^c^ |  |  |  | |  |  | | |  |  |  |  |  |  | |  |
|  | All learning community members learn from one anotherr.^1^ |  |  |  | |  |  | | |  |  |  |  |  |  | |  |
|  | The learning community uses input from various learning community members to gather new solutions.^2^ |  |  |  | |  |  | | |  |  |  |  |  |  | |  |
|  | The learning community members ensure that the learning community continues after the end of the project period.^1^ |  |  |  | |  |  | | |  |  |  |  |  |  | |  |

Space for explanation

…………………………………………………………………………………………………………………………………………………………….…………………………………………………………………………………………………………………………………………………………….…………………………………………………………………………………………………………………………………………………………….…………………………………………………………………………………………………………………………………………………

| **Learning community outputs** | |  | | **0 (absent / not applicable)** | | **1 (strongly disagree)** | | | **2** | **3** | **4** | **5** | **6** | **7** | **8** | **9** | **10 (strongly agree)** | |
| --- | --- | --- | --- | --- | --- | --- | --- | --- | --- | --- | --- | --- | --- | --- | --- | --- | --- | --- |
|  | Because of the learning community, I come up with improvement actions.^1^ | |  |  |  | |  |  | | |  |  |  |  |  |  | |  |
|  | The learning community meeting helps me to make the necessary adjustments in the current healthy weight approach.^1^ | |  |  |  | |  |  | | |  |  |  |  |  |  | |  |
|  | Because of the learning community meeting, I am able to adjust my approach to promoting healthy weight as needed.^2^ | |  |  |  | |  |  | | |  |  |  |  |  |  | |  |
|  | Formulating actions during the learning community meeting helps me sharpen how we want to achieve our goal together.^2, a^ | |  |  |  | |  |  | | |  |  |  |  |  |  | |  |
|  | I have the impression that the changes made by other learning community participants in their work complement my own changes.^2^ | |  |  |  | |  |  | | |  |  |  |  |  |  | |  |
|  | There is agreement on mission and purpose within the learning community.^1^ | |  |  |  | |  |  | | |  |  |  |  |  |  | |  |
|  | In the learning community, an increasingly concrete shared goal and vision is developed.^2,a^ | |  |  |  | |  |  | | |  |  |  |  |  |  | |  |
|  | The learning community members succeed in spurring others to take actions.^1^ | |  |  |  | |  |  | | |  |  |  |  |  |  | |  |
|  | The learning community meeting strengthens the cooperation among participating municipalities.^1^ | |  |  |  | |  |  | | |  |  |  |  |  |  | |  |
| **Intentions and actions after the learning community** | | |  |  |  | |  |  | | |  |  |  |  |  |  | |  |
|  | Thanks to my participation in the learning community, I am able to improve the healthy weight approach.^2^ | |  |  |  | |  |  | | |  |  |  |  |  |  | |  |
|  | I will apply what I have learned in daily practice.^1^ | |  |  |  | |  |  | | |  |  |  |  |  |  | |  |
|  | The learning community meeting helped me to generate new ideas about the healthy weight approach.^2^ | |  |  |  | |  |  | | |  |  |  |  |  |  | |  |

Space for explanation

…………………………………………………………………………………………………………………………………………………………….…………………………………………………………………………………………………………………………………………………………….…………………………………………………………………………………………………………………………………………………………….…………………………………………………………………………………………………………………………………………………

What is the most important output that you received today? For example, consider insights, ideas, documents, or resources. Why do you think this is useful?
…………………………………………………………………………………………………………………………………………………………….…………………………………………………………………………………………………………………………………………………………….…………………………………………………………………………………………………………………………………………………………….…………………………………………………………………………………………………………………………………………………

What will you specifically do with this output ? Write down your action. What can it set in motion that would not have happened without this output?
…………………………………………………………………………………………………………………………………………………………….…………………………………………………………………………………………………………………………………………………………….…………………………………………………………………………………………………………………………………………………………….…………………………………………………………………………………………………………………………………………………

How did this output contribute to your goal and/or your organization's healthy weight approach goal?

…………………………………………………………………………………………………………………………………………………………….…………………………………………………………………………………………………………………………………………………………….……………………………………………………………………………………………………………………………………………………

…….……………………………………………………………………………………………………………………………………………………

Footnotes:

*^1^ Participants rated these items according to a 6-point scale during LC meeting 2 and LC meeting 3: 1 = “no, definitely not”; 2 = “no, I do not think so”; 3 = “maybe”; 4 = “yes, I think so”; 5 = “yes, definitely”; and the additional option “do not know / not applicable”.*

*^2^ Participants rated these items according to a 11-point scale during LC meeting 2 and LC meeting 3: 1 (completely disagree) to 10 (completely agree). From LC meeting 4 onwards all items were rated according to the aforementioned 11-point scale including the additional option: “do not know / not applicable”. From LC meeting 6 onwards, all items were rated according to the abovementioned 11-point scale including the additional option “0 (absent / not applicable)”.*

*^a^ Item was missing within second LC meeting concept questionnaire. Item is rated according to footnote 2.*

*^b^ Item was missing in second and third LC meeting concept questionnaire. Item is rated according to footnote 2.*

*^c^ These three items were originally measured as one single item in the second and third LC meeting concept questionnaire. Item is rated according to footnote 2.*
