# Supplementary material for: The multidisciplinary and participatory process to develop the Rubric for Learning Communities about Health Approaches
Source: Front Public Health. 2025 Mar 5;13:1453197. doi: 10.3389/fpubh.2025.1453197 (PMC11919888; doi:10.3389/fpubh.2025.1453197)
Supplement: Supplementary file 5 [file Table_5.docx]

**Supplemental material 5: The final constructs and split half reliability coefficients based on LC meeting 4 (rubric version 4.2) and LC meeting 6 (rubric version 5)**

| **Final construct** | **Definition** | **Items** | **Split half LC meeting 6 (4)^4^** |
| --- | --- | --- | --- |
| Perceived cooperation LC^1^ | A safe and constructive LC where members cooperate, communicate openly, resolve conflicts constructively, and actively listen to one another. LC members are willing to compromise and function well as a team. These factors contribute to a positive learning and collaboration culture. | - The LC members cooperate well with one another. - The relationships among the LC members are strong. - The LC members deal with conflicts constructively. - The LC members have regular contact to keep one another informed and inspired. - The LC members are willing to compromise. - The LC members are open in their communication. - I am satisfied with the input of all LC members. - The LC members know how to find one another when something needs to be done. - The LC members actively listen to one another. - The LC functions well (structure, methods). | 0.97 (0.67) |
| Involvement approach^1^ | LC members feel an active desire and urgency to contribute to changing the healthy weight approach, even if it requires personal or organizational changes. | - I feel that strengthening the healthy weight approach is urgent. - I want to contribute to a change within the healthy weight approach, even if it requires a personal change and a change in my organization. - I feel very committed to finding solutions to strengthen the healthy weight approach. | 0.99  (0.63) |
| Involvement LC^1^ | LC members are actively involved, feel direct benefits, and are committed to the LC and its members. This includes creating goodwill and involvement within a member’s organization or department. | - I benefit my position/organization directly by participating in the LC. - I feel involved in the LC. - I believe that most LC members feel engaged in the LC. - I create goodwill and involvement for the LC within my organization/department. | 0.98  (0.74) |
| Learning from one another^2^ | The LC meeting creates an environment in which members can learn together on an individual and joint level by sharing knowledge and opinions with one another. The promotion of mutual reflection and collaboration results in new knowledge that ultimately leads to the identification of the next steps that can be taken to strengthen the healthy weight approach. | - All LC members learn from one another. - The LC made me realize my knowledge regarding strengthening the healthy weight approach. - The LC made me realize my knowledge gaps regarding strengthening the healthy weight approach. - The LC encourages me to share my opinion with others. - The LC encourages me to learn from others. - The LC uses input from various LC members to gather new solutions. - The people that I work with also learn from my involvement in the LC. - I now have a better understanding of the next steps that I can take to strengthen the healthy weight approach than I did before the LC meeting. | 0.99  (0.72) |
| Keep learning^2^ | The LC members are willing to continuously learn and take action to strengthen the healthy weight approach after the project period using the LC methods at individual and group level. Moreover, the LC members take mutual responsibility to gather and reflect on information about the healthy weight approach, as well as act upon newly acquired knowledge. | - I want to continue using the LC method after the project has ended. - The LC members ensure that the LC continues after the end of the project period. - I feel responsible for gathering information about the healthy weight approach. - I feel responsible for reflecting on information about the healthy weight approach. - I feel the need to do something with the newly acquired information about the healthy weight approach. | 0.96  (0.79) |
| LC output^2,3^ | LC outputs are perceived in terms of processes or products through shared goals and purposes, including resolutions to improve the healthy weight approach and strengthened cooperation. | - There is agreement on mission and purpose within the LC. - In the LC, an increasingly concrete shared goal and vision is developed. - The LC members succeed in spurring others to take actions. - The LC meeting helps me to make the necessary adjustments in the current healthy weight approach. - Because of the LC, I come up with improvement actions. - The LC meeting strengthens the cooperation among participating municipalities. - Formulating actions during the LC meeting helps me sharpen how we want to achieve our goal together. - Because of the LC meeting, I am able to adjust my approach to promoting healthy weight as needed. - I have the impression that the changes made by other LC participants in their work complement my own changes. | 0.93  (0.85) |
| Intentions^3^ | The LC members plan to apply their individually obtained knowledge and intended actions from the LC meeting in their daily practices to strengthen the healthy weight approach. | - Thanks to my participation in the LC, I am able to improve the healthy weight approach. - I will apply what I have learned in daily practice. - The LC meeting helped me to generate new ideas about the healthy weight approach. | 0.92  (0.92) |
| Network composition^1,2,3^ | All relevant healthy weight approach stakeholders are involved and participate actively in the LC because of LC members’ good collaboration and contact with the stakeholders. | - The LC involves the correct partners to achieve its purpose. - I involve healthy weight approach partners that are not LC members in what I learn. - The LC members are willing to include other healthy weight approach stakeholders in the LC meeting over time. - The LC meeting encourages me to reach out to relevant healthy weight approach partners. - The LC meeting helps to maintain the involvement of various healthy weight approach stakeholders. - The LC members have good contact with collaboration partners outside of the LC. - The LC meeting stimulates me to purposely collaborate with other stakeholders in the healthy weight approach. | 0.96  (0.72) |

^1^ = LC partnership experiences; ^2^ = learning; ^3^ = action; ^4^ The first number refers to the split half reliability coefficient of the final constructs as measured in rubric version 5 at LC meeting 6, and the second number (between brackets) refers to the final constructs as measured in rubric version 4.2 at LC meeting 4.
